# The Metabolic Functional Feature of Gut Microbiota in Mongolian Patients with Type 2 Diabetes

**DOI:** 10.4014/jmb.2402.02021

**Published:** 2024-04-29

**Authors:** Yanchao Liu, Hui Pang, Na Li, Yang Jiao, Zexu Zhang, Qin Zhu

**Affiliations:** 1 Department of Epidemiology, School of Public Health, Inner Mongolia Medical University, Inner Mongolia Autonomous Region, Hohhot 010110, P.R. China; 2 Laboratory for Molecular Epidemiology in Chronic Diseases, School of Public Health, Inner Mongolia Medical University, Inner Mongolia Autonomous Region, Hohhot 010110, P.R. China; 3 College of Continuing Education (IMAU Branch of Educational and Training Center for Central Agricultural Cadre), Inner Mongolia Agricultural University, Inner Mongolia Autonomous Region, Hohhot 010110, P.R. China

**Keywords:** Type 2 diabetes, gut microbiota, metabolites, Mongolian, metabolic pathway, *Clostridium* genus

## Abstract

The accumulating evidence substantiates the indispensable role of gut microbiota in modulating the pathogenesis of type 2 diabetes. Uncovering the intricacies of the mechanism is imperative in aiding disease control efforts. Revealing key bacterial species, their metabolites and/or metabolic pathways from the vast array of gut microorganisms can significantly contribute to precise treatment of the disease. With a high prevalence of type 2 diabetes in Inner Mongolia, China, we recruited volunteers from among the Mongolian population to investigate the relationship between gut microbiota and the disease. Fecal samples were collected from the Volunteers of Mongolia with Type 2 Diabetes group and a Control group, and detected by metagenomic analysis and untargeted metabolomics analysis. The findings suggest that *Firmicutes* and *Bacteroidetes* phyla are the predominant gut microorganisms that exert significant influence on the pathogenesis of type 2 diabetes in the Mongolian population. In the disease group, despite an increase in the quantity of most gut microbial metabolic enzymes, there was a concomitant weakening of gut metabolic function, suggesting that the gut microbiota may be in a compensatory state during the disease stage. β-Tocotrienol may serve as a pivotal gut metabolite produced by gut microorganisms and a potential biomarker for type 2 diabetes. The metabolic biosynthesis pathways of ubiquinone and other terpenoid quinones could be the crucial mechanism through which the gut microbiota regulates type 2 diabetes. Additionally, certain *Clostridium* gut species may play a pivotal role in the progression of the disease.

## Introduction

Diabetes mellitus (DM), characterized by chronic hyperglycemia, has emerged as a significant public health challenge in the 21st century, imposing substantial burdens on both human health and socioeconomic development [1]. In the context of DM, type 2 diabetes mellitus (T2D) accounts for 90% of cases and is primarily attributed to genetic and environmental factors. Recent research indicates that T2D prevalence rates in China are at 11.2%, with Inner Mongolia exhibiting a higher rate of 19.9% [2]. The proportion of Inner Mongolia's population over the age of 35 with diabetes was found to be 17.2% in recent studies conducted in that region [3], showing that there is a higher prevalence rate of T2D among this population. Extensive research has been conducted to prevent and treat the disease, and increasingly, this research has established that the gut microbiota plays a pivotal role in the development of T2D [4]. Therefore, the gut microbiota has become an important target for prevention and treatment of T2D [4, 5]. Identifying a mechanism at the strain and molecular level was considered the next step towards disease management, based on correlations between gut microbiota and T2D [1]. It has been revealed that *Firmicutes* and *Bacteroidetes* are the two predominant phyla in the adult human gut, accounting for over 90% of the total microbial community [6]. This implies that certain strains belonging to the both phyla may have a higher likelihood of being the key strains influencing T2D. A recognized mechanism by which the gut microbiota modulates disease is through the production of metabolites, which serve as a communication channel between the intestinal flora and host [7, 8]. Certain strains of butyrate-producing bacteria have been shown to confer health benefits by producing short-chain fatty acids (SCFAs), such as butyrate, which can help reduce the risk of T2D [7, 8]. In this study, our objective was to identify key gut bacteria and their associated metabolites involved in the progression of T2D among the Mongolian population.

## Materials and Methods

### Population Investigation

Volunteers were recruited from among the population of Inner Mongolia, China, with participants including newly diagnosed T2D patients and a control group with normal blood glucose levels. The inclusion and exclusion criteria were consistent with our previous research, and the other tests conducted on our volunteers, including fasting plasma glucose (FPG), weight, height, waist circumference, hip circumference, diastolic blood pressure (DBP), and systolic blood pressure (SBP), were also identical to those in our prior study [9].

### Metagenomic Analysis

Fresh fecal samples collected from participants were preserved at a temperature of -80°C and subsequently sent to Wuhan Metware Biotechnology Company for DNA extraction and metagenomic analysis. The methodology employed was consistent with that used in our previous study.

### Untargeted Metabolomics Analysis

**Sample preparation and extraction.** Each frozen fecal sample was thawed on ice and subsequently weighed 20 mg. Then, a solution containing internal standard (methanol:water = 7:3, v/v) was added at a volume of 400 μl and vortexed for 3 min. This mixture was sonicated in an ice bath for 10 min, followed by vortexing for 1 min. Subsequently, it was placed at -20°C for 30 min, and then centrifuged at 12,000 ×*g* for 10 min at 4°C. After removing the sediment, the supernatant was centrifuged at 12,000 ×*g* for 3 min at 4°C, and a 200 μl aliquot of the supernatant was transferred for LC-MS analysis.

### HPLC Conditions (T3)

All samples were obtained using the LC-MS system in accordance with machine protocols. The analytical parameters were set as follows: The column used was the Waters ACQUITY UPLC HSS T3 C18 (1.8 μm, 2.1 mm × 100 mm); the column temperature was maintained at 40°C; the flow rate was 0.4 ml/min; the injection volume was 2 μl; solvent system, water (0.1% formic acid); acetonitrile (0.1% formic acid); gradient program, 95:5 v/v at 0 min, 10:90 v/v at 11.0 min, 10:90 v/v at 12.0 min, 95:5 v/v at 12.1 min, 95:5 v/v at 14.0 min.

### Metabolites Analysis

The significantly regulated metabolites between two groups were identified based on the criteria of VIP >= 1, absolute Log2FC (fold change) >= 1, and *p*-value < 0.05. In addition, OPLS-DA (Orthogonal Partial Least Squares-Discriminant Analysis) was employed to extract VIP values, with the result also containing score plots and permutation plots, by using R package MetaboAnalystR. The data underwent log transformation (log2) and mean centering prior to OPLS-DA analysis. To prevent overfitting, a permutation test with 200 iterations was conducted.

The identified metabolites were annotated by referencing the KEGG Compound database (http://www.kegg.jp/kegg/compound/) and subsequently mapped to the KEGG Pathway database (http://www.kegg.jp/kegg/pathway.html). The hypergeometric test's *p*-value is utilized to identify significantly enriched pathways from a given list of metabolites.

### Origin Analysis of Differential Metabolites

The differential gut microorganisms and metabolites between groups have been uploaded to MetOrigin (http://metorigin.met-bioinformatics.cn/), an integrative database that includes seven different metabolite databases, including KEGG. This database provides information on the origin of the metabolites [10]. Additionally, the biological and statistical correlation between gut bacteria and metabolites will also be elucidated.

### Statistical Analysis

**Fig. 1:**
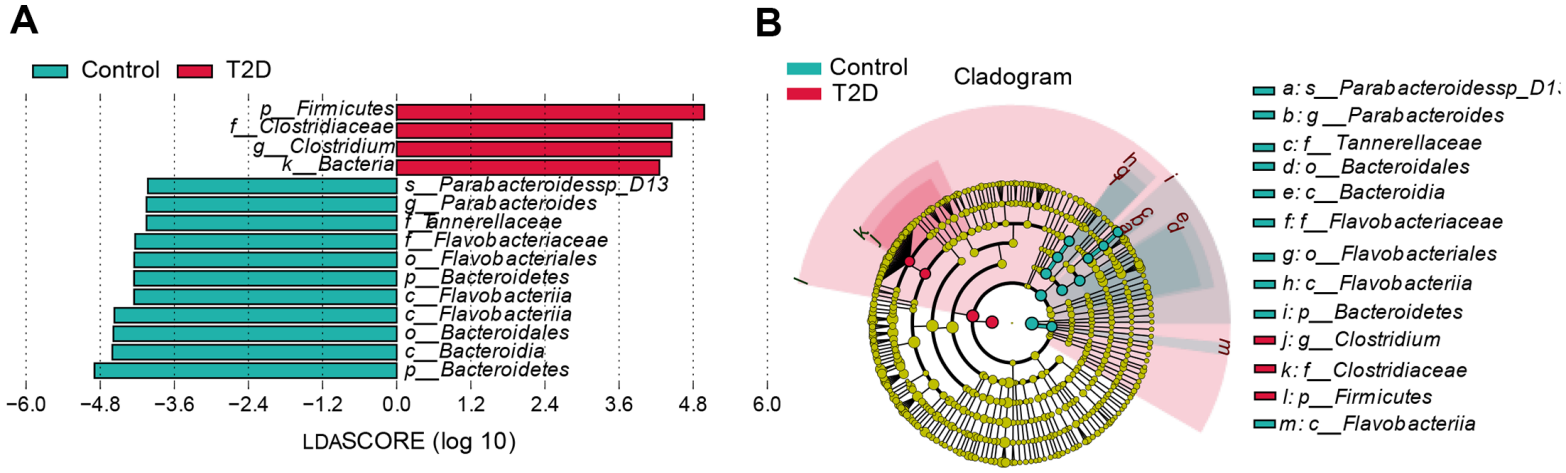
Results of the LEfSe analysis. (**A**) Distribution diagram illustrating the LDA scores of various species. The abscissa represented the LDA score, with red bars indicating microorganisms in the T2D group and green bars representing those in the Control group. (**B**) The phylogenetic tree depicting the evolutionary relationships among different species, and the circular pattern, extending from the center outwards, depicts the taxonomic hierarchy ranging from phylum to species. The red nodes represented the microbial flora that played a significant role in the red group, while the green ones signified those of importance in the green group.

**Fig. 2:**
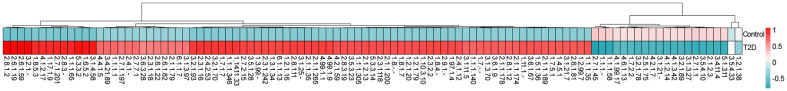
Cluster heatmap comparing the abundance of 103 metabolic enzymes between individuals in the T2D and Control groups. The red color indicates an increase in enzyme abundance, while blue represents a decrease. The intensity of the color reflects the quantity of enzymes present with deeper shades indicating higher or lower quantities.

The statistical analysis was conducted using SPSS26 and R software. The measurement data of a normal distribution were expressed as the mean ± SD, satisfying the requirements for parametric testing. Differences between groups were compared using Student's *t*-test. The data that did not satisfy the parameter test conditions were represented by median (interquartile range), and inter-group comparisons were analyzed using Kruskal-Wallis test. The chi-square test was employed to analyze count data. Statistically significant differences were observed when the level of significance was set at *p < 0.05, **p < 0.01, and ***p < 0.001. The completion of Figs. 1 and 2 was facilitated by utilizing Wekemo Bioincloud (https:// www.bioincloud.tech).

## Results

### Participant Characteristics

Out of 160 volunteers, only 17 qualified participants were selected based on the inclusion and exclusion criteria. These individuals were then divided into two groups: a diabetes group (T2D) consisting of five cases with FPG levels ≥7.0 mmol/l (126 mg/dl), which meets with the diagnostic standard for T2D recommended by World Health Organization; and a normal glucose control group (Control) consisting of twelve cases with FPG levels < 6.0 mmol/l. The male-to-female sex ratio in the T2D group was 2:3, while that in the Control group was 5:7. There was no statistically significant difference in the sex ratio between the two groups according to Fisher’s exact probability (*p* = 1.000). The study was designed as a matching case-control study; each T2D case was matched with 2-3 control cases of the same gender and similar age. The waist circumference and waist-to-hip ratio (WHR) of the participants exhibited statistically significant differences between the T2D and Control groups, with T2D patients displaying higher values for both measures than those in the Control group (Table 1). The comprehensive information on each participant is presented in Table S1.

### Differential Gut Microbial Species Identified by Metagenomic Analysis Between T2D and Control Groups

The relative abundance of the seven taxonomic levels, including kingdom, phylum, class, order, family, genus, and species were analyzed. Four kingdoms, *Bacteria*, *Viruses*, *Archaea*, and *Eukaryota*, and 120 phyla, 101 classes, 210 orders, 433 families, 1526 genera, and 6638 species from fecal samples of the participants were detected. Although Principal Component Analysis (PCA) [11] and Non-Metric Multidimensional Scaling (NMDS) [12] were employed to assess the diversity differences among groups at each level, no statistically significant differences were observed. However, there were significant variations observed in the bacterial composition at both genus and species levels between the group with T2D and those without. In the bacteria, the genus *Blautia* with the highest quantity was dominant, and it was higher in T2D than in Control. However, the number of most different bacteria was higher in Control than in T2D, but Control had more abundance of microorganisms belonging to the class *Flavobacteriia*. The detailed information on the different bacteria is shown in Table S2.

Next, we measured different species among groups by the rank-sum test, reduced the dimension by linear discriminant analysis (LDA) [13], and evaluated the influence of different species through the LDA score. The criterion was number 4, which meant that the bacteria significantly impacted the group when its LDA score was over 4. It was evident that the microorganisms with a higher LDA score in the T2D group all belonged to the *Firmicutes* phylum, especially the *Clostridium* genus, and that in Control, they all belonged to the *Bacteroidetes* phylum, especially the *Flavobacteriia* and *Bacteroidia* classes (Fig. 1).

### Comparative Analysis of Metabolic Enzyme Abundance by Metagenomic Analysis

After searching the KEGG database and conducting the statistical analysis, we found that 103 metabolic enzymes of gut microbiota exhibited significant differences in relative abundance between groups, with 80% of them increased in the T2D group (Fig. 2).

### Comparison of Metabolic Profiles Between T2D and Control Groups by Untargeted Metabolomics Analyses

**Fig. 3:**
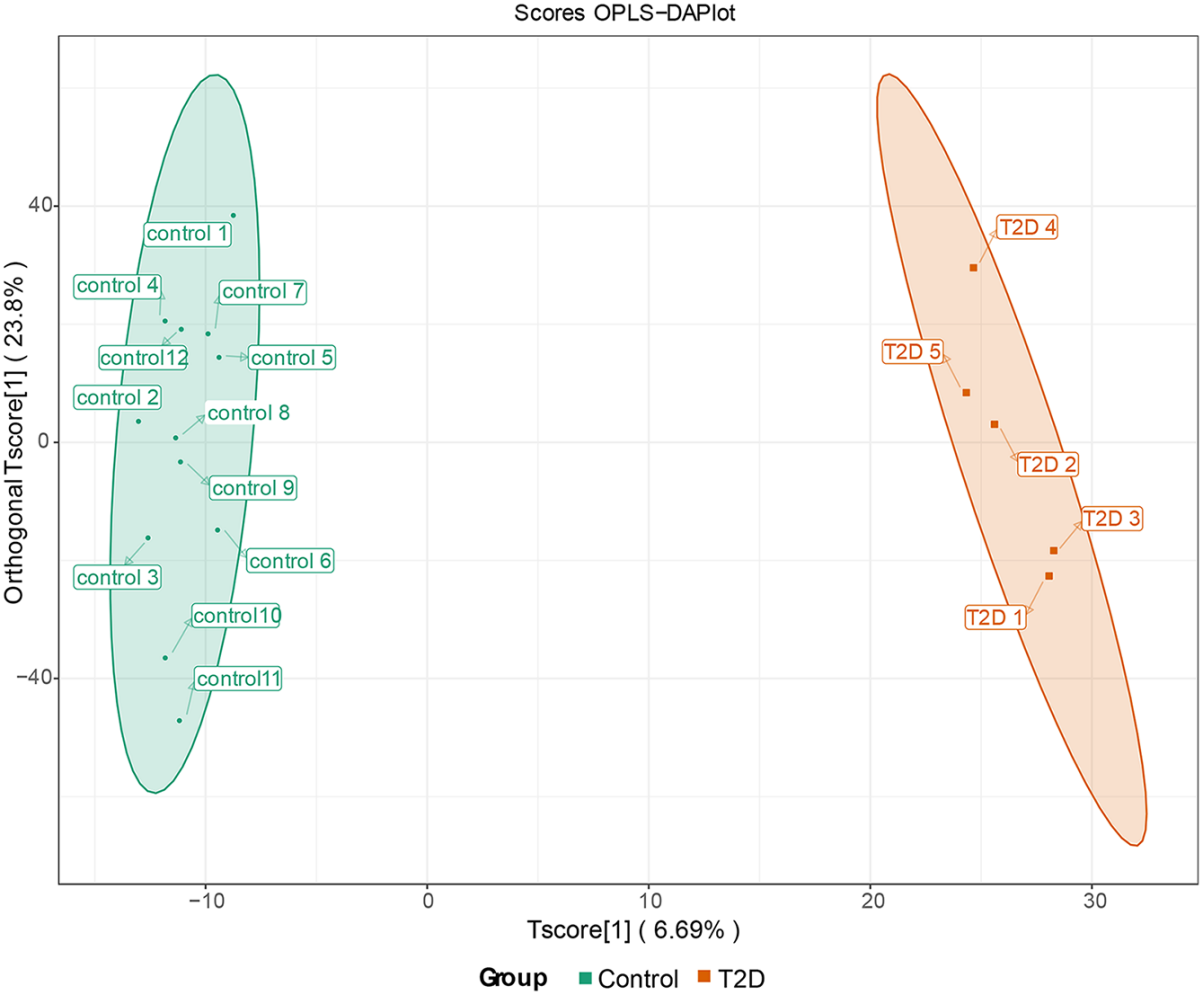
The OPLS-DA analysis revealed significant differences in metabolite profiles between the T2D and Control groups. The abscissa represented the score value of the prediction component, and the difference between groups could be seen in the abscissa direction. The ordinate represented the score value of the orthogonal component, and the ordinate direction could be seen in the difference within the group. Percentage represented the extent of explanation of components to the data set.

A total of 4,597 metabolites were detected, which were mainly amino acid and its metabolites, benzene and substituted derivatives, heterocyclic compounds, aldehyde, ketones, esters, and organic acid and its derivatives. We analyzed the difference of metabolites between groups by OPLS-DA [14] and a significant difference was shown (Fig. 3). Parameters for the OPLS-DA model evaluation were: R^2^Y=0.993, Q^2^=0.318 and R^2^X=0.441.

### Differential Metabolites Observed in the T2D Group

**Fig. 4:**
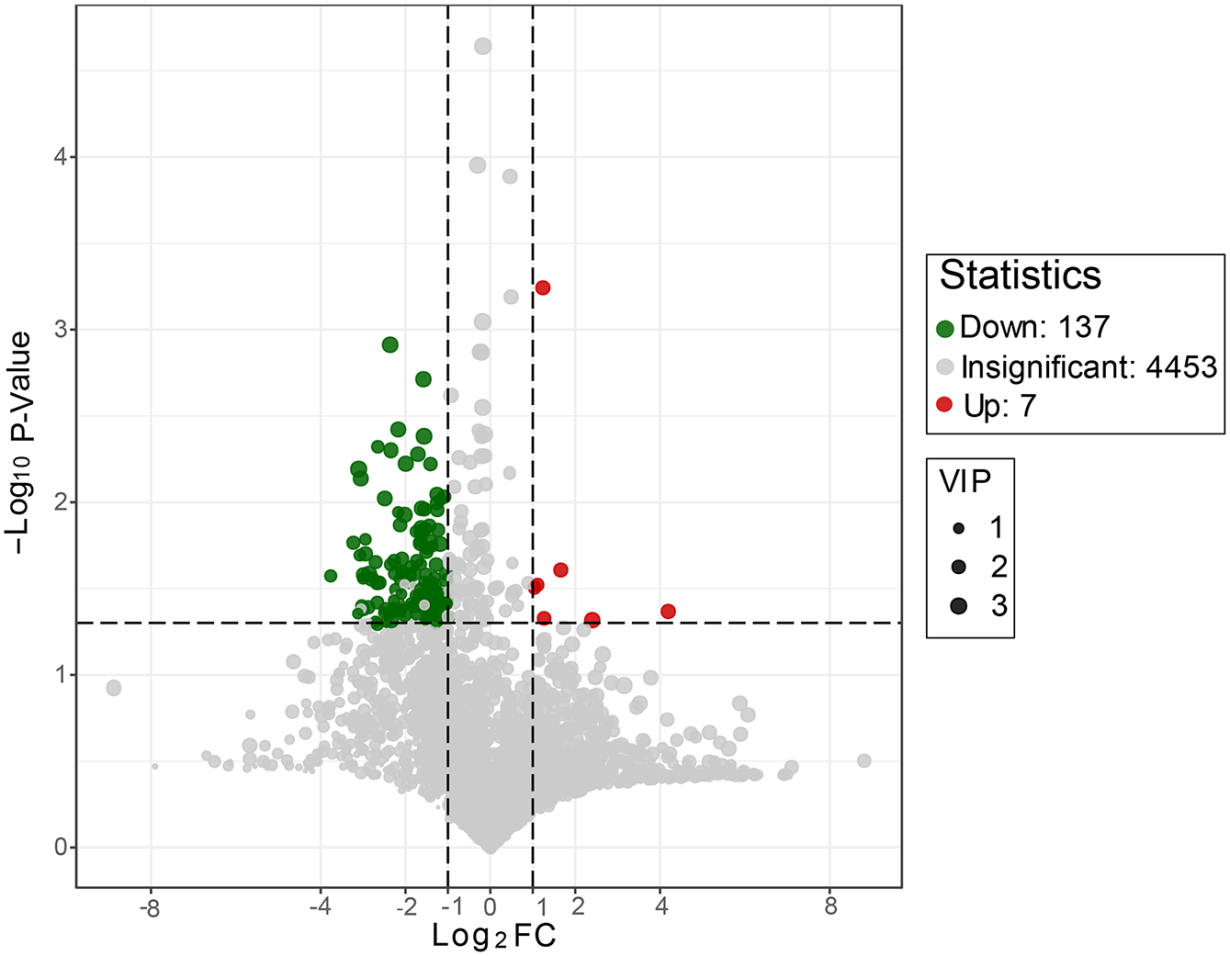
The Volcano Plot result of the differential abundance of metabolites in the T2D group compared to the Control group. Green and red spots presented the significant metabolites between the two groups. Green meant decreasing but red meant increasing, and the gray ones were the metabolites with no obvious change.

There were 144 metabolites showing significant difference between the two groups in relative abundance, including 100 positive ions and 44 negative ions. Compared to the Control group, in the T2D group, there were 137 gut metabolites reduced including 97 positive ions and 40 negative ions, and only 7 raised including three positive ions and four negative ions (Fig. 4). The detail information on all the differential metabolites was described in Table S3 and the information on every sample can be found in Table S4. This result was the inverse of that of metabolic enzymes, which may reflect a compensatory mechanism of the disordered microbiota in that the metabolic function of the gut decreased in the T2D group.

### Origin Analysis Revealed the Various Sources of the Differential Metabolites

**Fig. 5:**
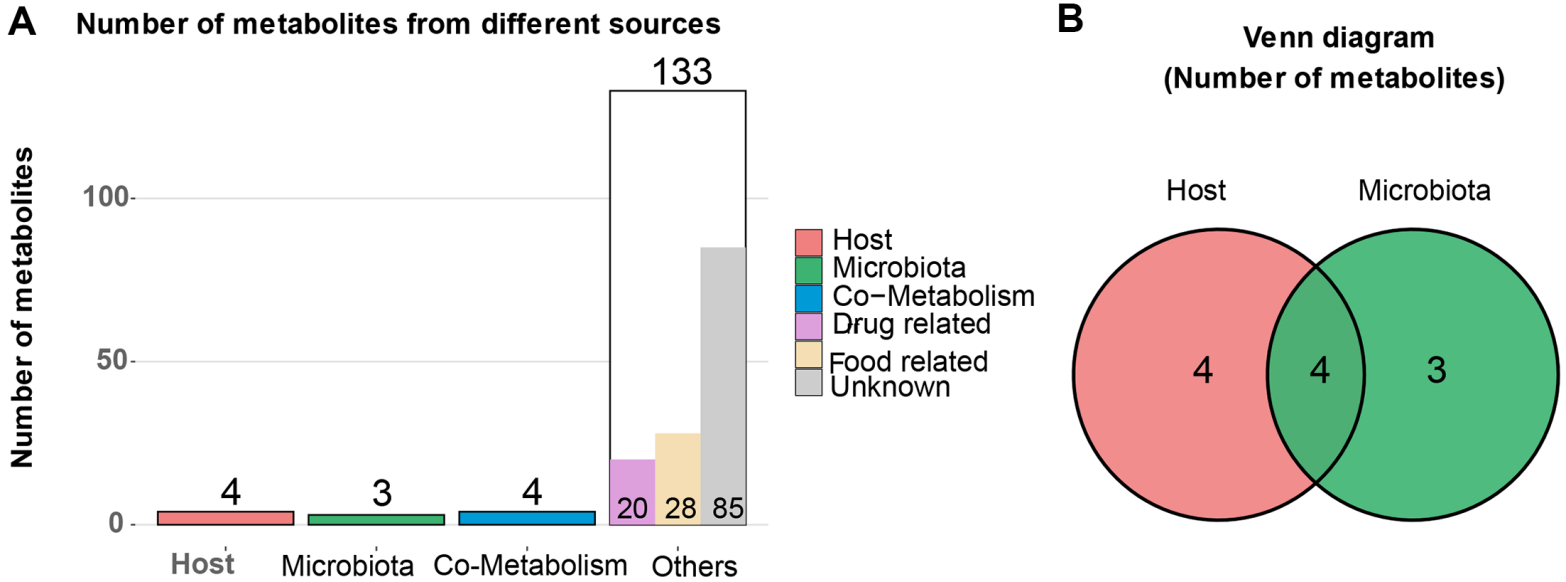
The results of the origin analysis of differential metabolites. (**A**) The total number of metabolites from different sources. (**B**) The number of metabolites from intestinal microorganism, host, or co-metabolism.

By using MetOrigin, we found that eleven metabolites originated from intestinal microorganisms or host, with three metabolites, fusidate sodium, 3-hydroxyphenylacetic acid, and β-tocotrienol coming from intestinal microorganisms, four originating from host, and another four originating from co-metabolism (produced by host and microorganism). Fig. 5 depicts information on the eleven metabolites listed below in Table 2, all of which were decreased in the T2D group.

The detailed information on all the results can be found in Table S3.

### Enrichment Analysis of Metabolic Pathways

The differential metabolites between groups were enriched in three metabolic pathways. Ubiquinone and other terpenoid quinone biosynthesis was from intestinal microorganism, while arachidonic acid metabolism and steroid hormone biosynthesis were both from host. In the arachidonic acid metabolism pathway, there were four metabolites decreased in the T2D group, which were prostaglandin I2, leukotriene A4, 15-keto-prostaglandin F2a, and Trioxilin B3. In the steroid hormone biosynthesis pathway, the 5alpha-dihydrodeoxycorticosterone decreased in the T2D group.

### Biological and Statistical Correlation Analysis of Ubiquinone and Other Terpenoid Quinone Biosynthesis Pathways

**Fig. 6:**
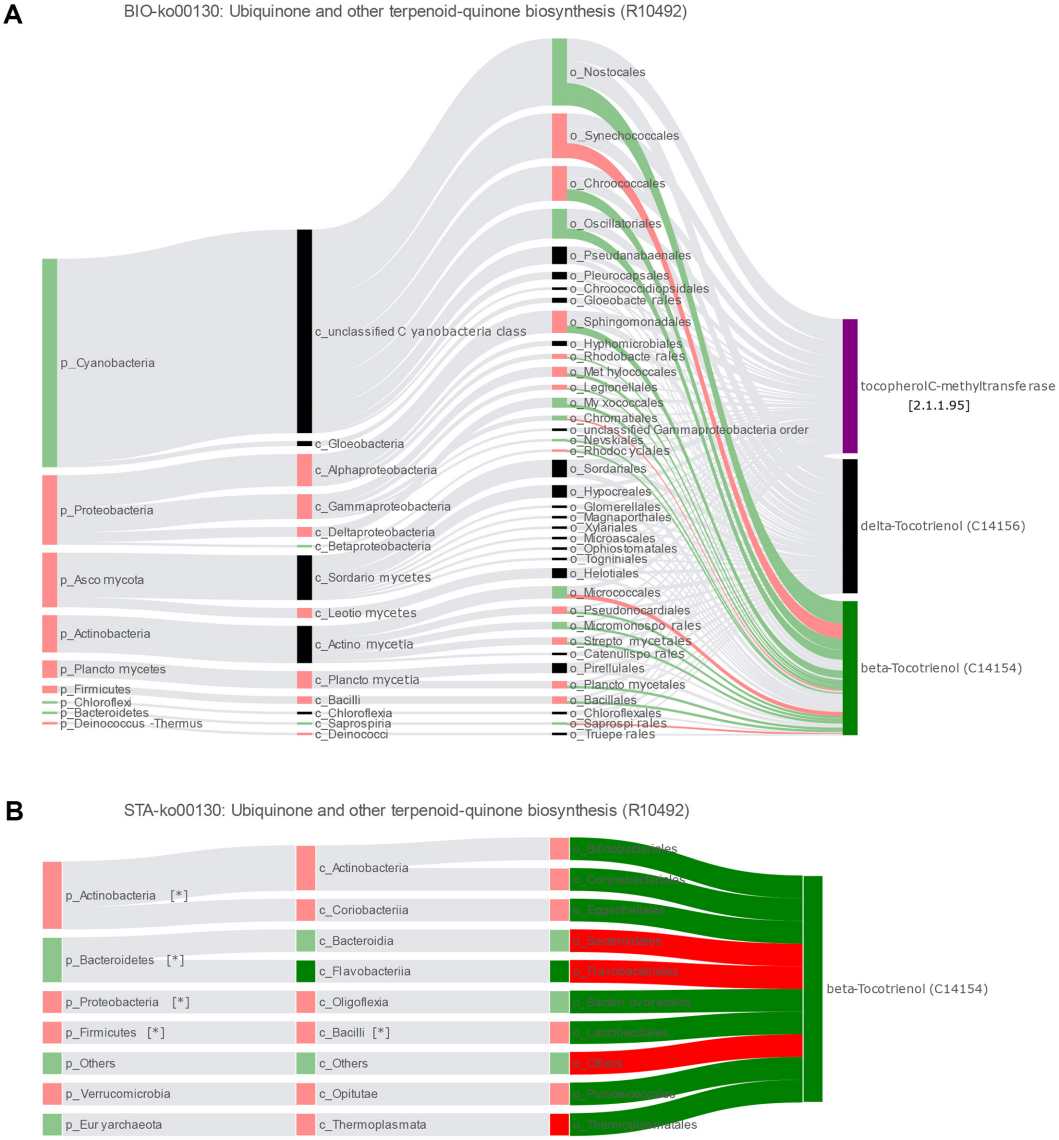
Sankey Networks demonstrated the results of correlation between the gut microbiota and metabolites in a certain metabolic pathway. (**A**) A BIO-Sankey Network showed all the microbiome that biologically correlated to the β-tocotrienol in the ubiquinone and other terpenoid quinone biosynthesis pathways. Dark red bars meant significantly upregulated microbes or metabolites (FC > 1 and *p* < 0.05) in the T2D group; light red bars meant upregulated microbes or metabolites (FC > 1 and *p* ≥ 0.05) in the T2D group; dark green bars meant significantly downregulated microbes or metabolites (FC < l and *p* < 0.05) in the T2D group; light green bars meant downregulated microbes or metabolites (FC < 1 and *p* ≥ 0.05) in the T2D group; dark grey bars meant microbes or metabolites with no change (FC = 1) in the two groups; black bars meant microbes or metabolites in the reference database; purple bars meant metabolic enzymes; dark red bands meant significant positive correlation (R > 0 and *p* < 0.05); light red bands meant positive correlation without statistical significances (R > 0 and *p* ≥ 0.05); dark green bands meant significant negative correlation (R < 0 and *p* < 0.05); light green bands meant negative correlation without statistical significance (R < 0 and *p* ≥ 0.05); dark gray bands meant no correlation (R = 0); and light gray bands meant reference relationships searched from database. (**B**) A STA(Statistical)-Sankey Network that summarized statistical correlations existed in microorganisms and β-tocotrienol in the ubiquinone and other terpenoid quinone biosynthesis pathways, in this study. *Meant difference with statistical significance.

Sankey Networks from MetOrigin described the biological and statistical correlation of gut microbiome and metabolites. In the ubiquinone and other terpenoid quinone biosynthesis pathways (https://www.kegg.jp/pathway/ko00130), the β-tocotrienol is produced by the catalysis of tocopherol C-methyltransferase, which converts delta-Tocotrienol and S-adenosyl-L-methionine. The β-tocotrienol decreased in T2D group and several gut bacteria had biological and statistical significant correlation with that. The *Firmicutes*, *Proteobacteria*, *Actinobacteria* phyla and the *Bacilli* class negatively correlated with the β-tocotrienol. Conversely, the *Bacteroidetes* phylum positively correlated with that (Fig. 6).

### Correlation Analysis of Gut Bacteria and Metabolites

We analyzed the statistical correlation between the gut microorganisms and metabolites in all metabolic pathways using Spearman’s nonparametric inertia analysis. We found out that many species belonging to *Clostridium* genus were negatively correlated to most metabolites, including β-tocotrienol (Fig. S1).

## Discussion

The mechanisms of gut microbiota regulating T2D are complicated [15], and the metabolites from these gut bacteria are no doubt the important mediates and substrate. Not only can the gut microbiome be the biomarker of T2D, but the metabolites from the gut microorganisms also have that capacity [16]. The metabolites from fecal sample consist of several sources, including food, medicine, host, microorganisms, and others [10]. In fact, most metabolites are from unknown sources, and more research is needed to identify them. Since metabolites from food and host are easily affected by diet, as constituents of the microbiome they are more important in reflecting the state of both host and gut microbiota. β-Tocotrienol is a kind of vitamin E [17] and a product of s-adenosyl-l-methionine and delta-tocotrienol. A cohort study demonstrates a negative correlation between the intake of β-tocotrienol and the risk of developing T2D [18], which is consistent with our findings. In addition, another study proved that delta-tocotrienol can reduce inflammation in systemic and adipose tissues to improve T2D [19]. The evidence shows that both product β-tocotrienol and substrate delta-tocotrienol are beneficial for glycemic control. Interestingly, research from Japan proved an inverse relationship between the *Blautia* genus and T2D [20], which contradicts our study. We suppose that the difference may be attributed to the varying diets between Mongolians and Japanese. Considering that *Blautia wexlerae*, which produces s-adenosylmethionine, can ameliorate T2D, the long-term insufficient intake of delta-tocotrienol in Mongolian T2D patients contributes to compensatory increases in the population of *Blautia* genus bacteria that produce s-adenosylmethionine. However, this hypothesis needs to be proven in the future.

Fusidate Sodium (Fusidin) is an antibiotic used mainly for the treatment of *Staphylococcus* infections [21]. In an animal experiment, Fusidin could ameliorate the course of diabetes [22]. Although some other studies have shown conflicting results on improving diabetes with antibiotics [23], a more in-depth study to uncover the function of Fusidin that regulates diabetes. In addition, 3-hydroxyphenylacetic acid is an intermediate metabolite of quercetin metabolized by the intestinal microbiome [24], and lots of studies proved that quercetin has a function to treat T2D [25]. This implies that 3-hydroxyphenylacetic acid is a potential key metabolite affecting the course of T2D.

Our previous study revealed the importance of gut *Clostridium* genus in T2D, and the hypothesis has been proved by this multi-omics study. The *Clostridium* genus consists of many species, including pathogenic bacteria like *C. hathewayi* and *C. symbiosum*, as well as butyrate-producing bacteria such as *Clostridium* cluster XIVa and *C. indolis* [26]. The *Clostridium* genus also strongly affected protein hydrolysis [27], and moreover, we found out that several species belonging to the genus are significantly associated with β-tocotrienol. All of this evidence supports that the *Clostridium* genus is an important source of key gut microbial strains that interfere in the development of T2D. Although the current data from relevant databases are not able to support the bio-association between these *Clostridium* species and β-tocotrienol, the research offers clues to discover the key gut bacteria and metabolic pathway in regulating T2D.

The present study admittedly suffers from a sampling error due to the small size of samples, and further investigation is required to validate the results.

## Supplemental Materials

Supplementary data for this paper are available on-line only at http://jmb.or.kr.





## Figures and Tables

**Table 1:** Characteristics of the participants in both groups.

Group	T2D	Control	*t* value	*p* value
FPG, mmol/l	11.58 ± 5.83	5.52 ± 0.29	3.77	0.00
Age, years	60.80 ± 8.50	55.08 ± 7.79	1.35	0.20
BMI	27.96 ± 2.38	24.97 ± 3.44	1.76	0.10
Waistline, cm	102.60 ± 6.80	88.46 ± 7.44	3.65	0.00
Hipline, cm	105.00 ± 6.44	99.58 ± 7.01	1.48	0.16
WHR	0.98 ± 0.33	0.89 ± 0.42	4.22	0.00
SBP, mmHg	133.80 ± 18.73	129.92 ± 17.30	0.73	0.48
DBP, mmHg	83.60 ± 13.26	85.50 ± 11.34	-0.30	0.77

**Table 2:** Information on the eleven metabolites from intestinal microorganism or host or co-metabolism.

HMDBID	KEGGID	Name	Origin
HMDB0015570	C06694	Fusidate Sodium	Microbiota
HMDB0000440	C05593	3-Hydroxyphenylacetic acid	Microbiota
HMDB0030554	C14154	beta-Tocotrienol	Microbiota
HMDB0006845	C15776	4alpha-Methylfecosterol	Co-Metabolism
HMDB0000619	C00695	cholic acid	Co-Metabolism
HMDB0001337	C00909	Leukotriene A4	Co-Metabolism
HMDB0012453	C17333	3beta-Hydroxy-5-cholestenoic acid	Co-Metabolism
HMDB0000879	C13713	Tetrahydrodeoxycorticosterone	Host
HMDB0001335	C01312	Prostaglandin I2	Host
HMDB0060407	C18040	5alpha-Dihydrodeoxycorticosterone	Host
HMDB0004026	C05485	21-Hydroxypregnenolone	Host
