# Supplementary figures and images for: The Metabolic Functional Feature of Gut Microbiota in Mongolian Patients with Type 2 Diabetes
Source: J Microbiol Biotechnol. 2024 Apr 29;34(6):1214–21. doi: 10.4014/jmb.2402.02021 (PMC11239439; doi:10.4014/jmb.2402.02021)

Correlation heatmap

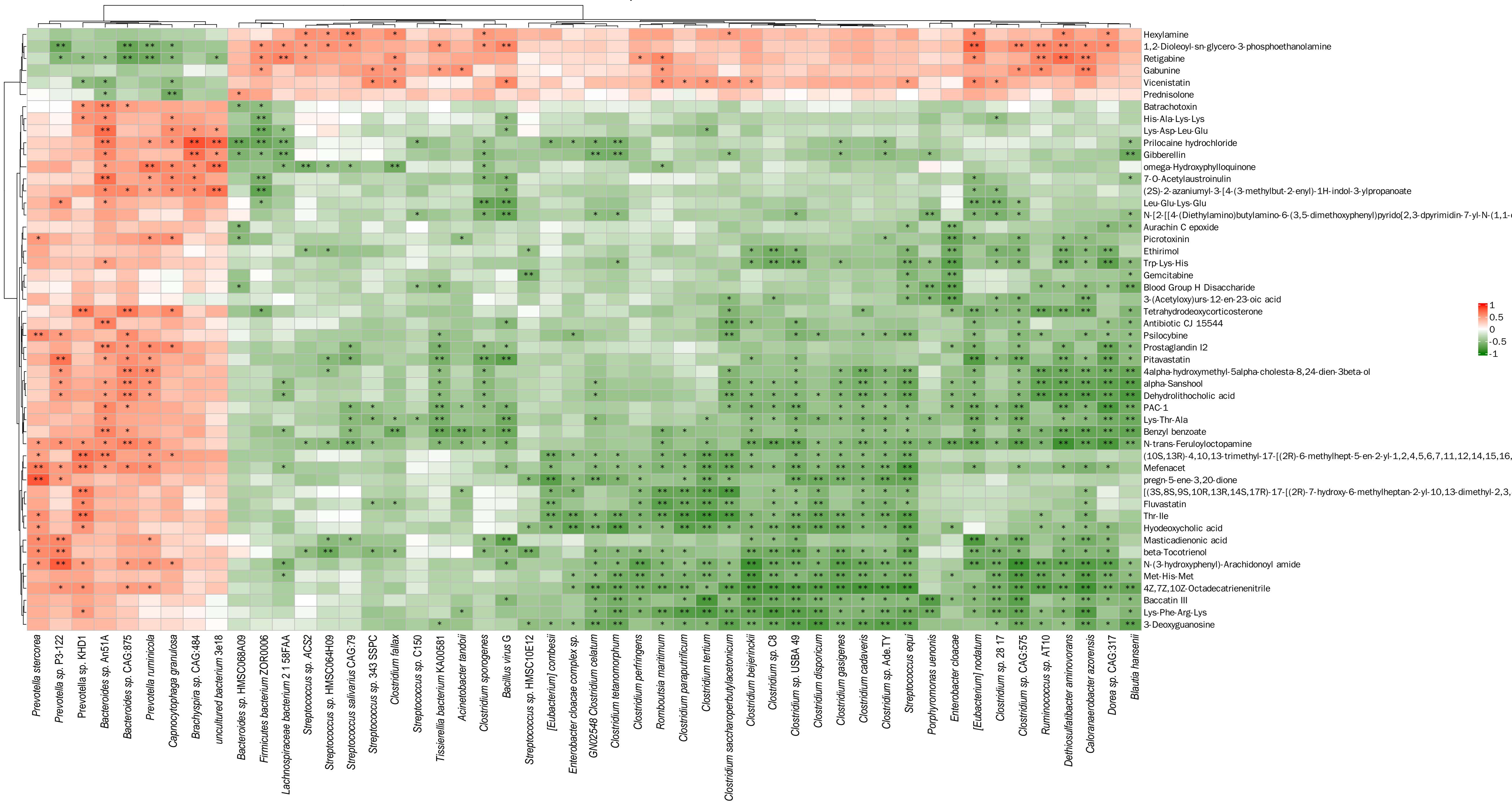

Supplement: Supplementary file 1 [file jmb-34-6-1214-supple1.pdf]
